# Blood platelet RNA enables the detection of multiple sclerosis

**DOI:** 10.1177/2055217320946784

**Published:** 2020-07-30

**Authors:** Nik Sol, Cyra E Leurs, Sjors GJG In ’t Veld, Eva M Strijbis, Adrienne Vancura, Markus W Schweiger, Charlotte E Teunissen, Farrah J Mateen, Bakhos A Tannous, Myron G Best, Thomas Würdinger, Joep Killestein

**Affiliations:** Department of Neurology, Neuroscience Amsterdam, VUmc MS Center Amsterdam, Amsterdam UMC, VU University Medical Center, Amsterdam, The Netherlands; Brain Tumor Center Amsterdam, Amsterdam UMC, VU University Medical Center, Amsterdam, the Netherlands; Department of Neurology, Neuroscience Amsterdam, VUmc MS Center Amsterdam, Amsterdam UMC, VU University Medical Center, Amsterdam, The Netherlands; Brain Tumor Center Amsterdam, Amsterdam UMC, VU University Medical Center, Amsterdam, the Netherlands; Department of Neurosurgery, Amsterdam UMC, VU University Medical Center, Amsterdam, the Netherlands; Department of Neurology, Neuroscience Amsterdam, VUmc MS Center Amsterdam, Amsterdam UMC, VU University Medical Center, Amsterdam, The Netherlands; Brain Tumor Center Amsterdam, Amsterdam UMC, VU University Medical Center, Amsterdam, the Netherlands; Department of Neurosurgery, Amsterdam UMC, VU University Medical Center, Amsterdam, the Netherlands; Brain Tumor Center Amsterdam, Amsterdam UMC, VU University Medical Center, Amsterdam, the Netherlands; Department of Neurosurgery, Amsterdam UMC, VU University Medical Center, Amsterdam, the Netherlands; Department of Neurology, Massachusetts General Hospital Harvard Medical School, Boston, MA, USA; Neurochemistry Laboratory and Biobank, Department of Clinical Chemistry, Neuroscience Campus Amsterdam, Amsterdam UMC, VU University Medical Center, Amsterdam, The Netherlands; Department of Neurology, Massachusetts General Hospital Harvard Medical School, Boston, MA, USA; Brain Tumor Center Amsterdam, Amsterdam UMC, VU University Medical Center, Amsterdam, the Netherlands; Department of Neurosurgery, Amsterdam UMC, VU University Medical Center, Amsterdam, the Netherlands; Department of Pathology, Amsterdam UMC, VU University Medical Center, Amsterdam, the Netherlands; Brain Tumor Center Amsterdam, Amsterdam UMC, VU University Medical Center, Amsterdam, the Netherlands; Department of Neurosurgery, Amsterdam UMC, VU University Medical Center, Amsterdam, the Netherlands; Department of Neurology, Neuroscience Amsterdam, VUmc MS Center Amsterdam, Amsterdam UMC, VU University Medical Center, Amsterdam, The Netherlands

**Keywords:** Multiple sclerosis, platelets, biomarker, RNA, diagnostics

## Abstract

**Background:**

In multiple sclerosis (MS), clinical assessment, MRI and cerebrospinal fluid are important in the diagnostic process. However, no blood biomarker has been confirmed as a useful tool in the diagnostic work-up.

**Objectives:**

Blood platelets contain a rich spliced mRNA repertoire that can alter during megakaryocyte development but also during platelet formation and platelet circulation. In this proof of concept study, we evaluate the diagnostic potential of spliced blood platelet RNA for the detection of MS.

**Methods:**

We isolated and sequenced platelet RNA of blood samples obtained from 57 MS patients and 66 age- and gender-matched healthy controls (HCs). 60% was used to develop a particle swarm-optimized (PSO) support vector machine classification algorithm. The remaining 40% served as an independent validation series.

**Results:**

In total, 1249 RNAs with differential spliced junction expression levels were identified between platelets of MS patients as compared to HCs, including EPSTI1, IFI6, and RPS6KA3, in line with reported inflammatory signatures in the blood of MS patients. The RNAs were subsequently used as input for a MS classifier, capable of detecting MS with 80% accuracy in the independent validation series.

**Conclusions:**

Spliced platelet RNA may enable the blood-based diagnosis of MS, warranting large-scale validation.

## Introduction

Multiple sclerosis (MS) is a chronic inflammatory demyelinating disorder affecting the central nervous system. Clinical assessment, magnetic resonance imaging (MRI), and cerebrospinal fluid (CSF) analysis play important roles in the diagnostic process.^1^ The field of MS biomarker discovery is thriving and the search for precise diagnostic tests continues. So far, no blood-based biomarker for MS has been confirmed.^2^ Minimally invasive blood-based biomarkers would complement current MS diagnostics and monitoring.

**Figure 1.**
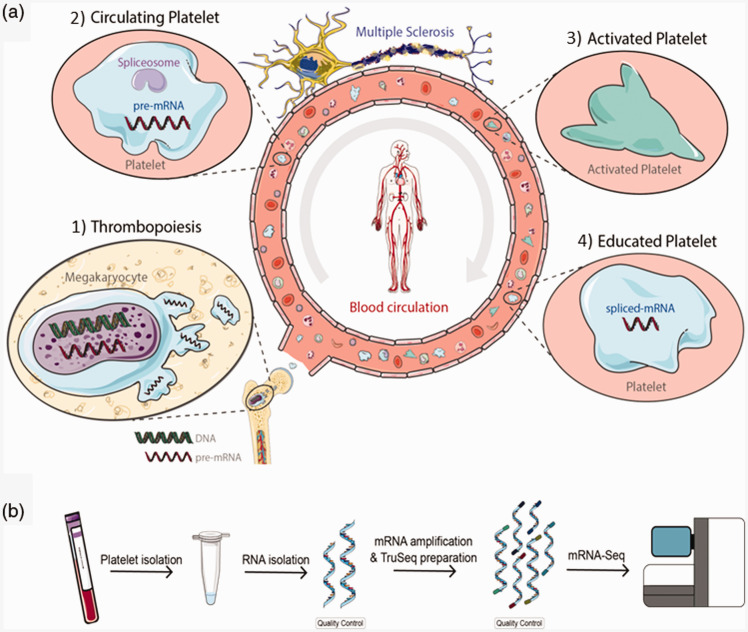
Schematic overview of platelet generation, circulation and possible alteration/education in the presence of MS. (a) 1) Blood platelets are generated from megakaryocytes residing in the bone marrow. During the final stages of thrombopoiesis, platelets are loaded with pre-mRNAs before budding from the megakaryocyte. 2) Circulating platelets respond to activating signals from their environment with specific splicing of their pre-mRNAs and uptake of RNA from different cell types. 3) Blood platelets in MS patients show increased levels of adhesiveness and activation. 4) Processes involved in MS could potentially lead to specific splicing of platelet RNA, resulting in a disease-specific RNA signature. (b) Schematic overview of the thomboSeq workflow. Blood was collected in 6 ml EDTA-coated tubes after which the platelet RNA is isolated, amplified, and labeled for sequencing. The RNA isolation and amplification steps are subjected to quality control using Bioanalyzer analysis.

During the final stages of thrombopoiesis, platelets are loaded with pre-mature messenger RNAs (pre-mRNAs) before being released from the megakaryocyte.^3^ As a result, platelets contain a rich RNA repertoire that can change during megakaryocyte development but also during platelet formation and platelet circulation (Figure 1(a)). Especially the change of RNA transcripts during circulation, possibly achieved by specific splicing queues, is of relevance in the present study. Platelets respond to activating signals from their environment with specific splicing of their pre-mRNAs and potential uptake of RNA from different cell types, leading to a unique and dynamic RNA repertoire.^4–6^ It has been shown that RNA isolated from tumor-educated platelets, platelets subjected to RNA changes in patients with cancer, may lead to highly accurate identification of traces of the tumor in blood,^7^ independent of inflammatory conditions.^4^

Blood platelets are able to participate in inflammatory responses by secreting different cytokines and interact with different cell types, such as leukocytes and vascular cells.^6^ Furthermore, platelets may be involved in the progression and pathogenesis of MS.^8,9^ It has been observed that platelets from MS patients exhibit high levels of activation.^10–12^ Interestingly, in vivo murine studies demonstrated that platelet depletion reduces MS disease severity.^12^ Furthermore, coagulation in which platelets play a major role, seems to also play a role in the pathogenesis of MS, suggesting that platelets could serve as target for new therapeutic approaches.^13^ As a result of their involvement in the immune response and potential role in the progression and development of the disease, we hypothesize that blood platelets of MS patients contain a disease-related RNA-signature which could be used as a diagnostic tool (Figure 1(a)).

## Results

### Platelet collection

Blood was collected from healthy controls (HCs) (n = 66) and MS patients (n = 57) in EDTA-coated Vacutainer tubes. All patients were diagnosed according to the revised McDonald criteria^1^ and had relapsing remitting MS for at least 10 years. Of the MS patients, 17 were male and 40 were female, resulting in a ratio of 1:2.4 (Table 1 and S1). All 66 HCs were age- and gender-matched. At the time of blood draw, the disability status of MS patients was examined according to the Kurtzke Expanded Disability Status Scale (EDSS) and an MRI scan was acquired. Although all patients were in clinical remission, 29 of them showed new T2-hyperintense lesions compared to an earlier performed MRI scan (Table 1).

**Table 1.** Patient characteristics.

	Healthy controls(n = 66)	Multiple Sclerosis (n = 57)	P value
Gender			
Male n(%)	22 (33)	17 (30)	0.70
Female n(%)	44 (67)	40 (70)	
Age (mean ± SD, year)	46.5 ± 7.4	46.6 ± 6.9	0.91
DMT n(%)	NA	27 (47)	
New T2 lesions n(%)	NA	29 (51)	
EDSS (mean ± SD)	NA	3.0 ± 0.9	

### Platelet RNA as a diagnostics tool for MS

Platelet RNA was sequenced and analyzed according to a previously published protocol^14^ (Figure 1(b)). Briefly, platelets were isolated from whole blood by differential centrifugation, platelets were lysed, and RNA was isolated. The RNA was subsequently subjected to RNA amplification and prepared for RNA-sequencing on the Illumina platform.

We employed our previously published thromboSeq classification software for algorithm development.^4,14^ First we randomly selected from the full dataset 17 MS and 19 HCs samples (30% of the total sample size) and assigned those to the training series, employed for biomarker RNA panel selection. Then, the algorithm optimized the biomarker panel towards accurate prediction by a readout of the randomly selected evaluation series (n = 17 MS and n = 20 HCs). This resulted in a total of 1,249 spliced RNAs which were found to be optimal for blood-based MS diagnostics (Table S2). Of this subset, 645 RNAs were increased in platelets of MS patients as compared to HCs, including the RNAs EPSTI1 and IFI6, whereas RPS6KA3 had decreased levels in MS patients as compared to HCs (Table 2; Table S2).

**Table 2.** Top RNAs with differentials spliced junctions.

	Up in MS	Down in MS
1	EPSTI1	HBB
2	DCUN1D4	AHCYL1
3	MTND1P23	RPS6KA3
4	IFI6	CDK16
5	MTND2P28	ADI1
6	UBE2L6	TADA3
7	MTRNR2L12	TMED4
8	MTND4P12	EFHC1
9	ATF7IP	AMPD2
11	MTATP6P1	TUBB

**Figure 2.**
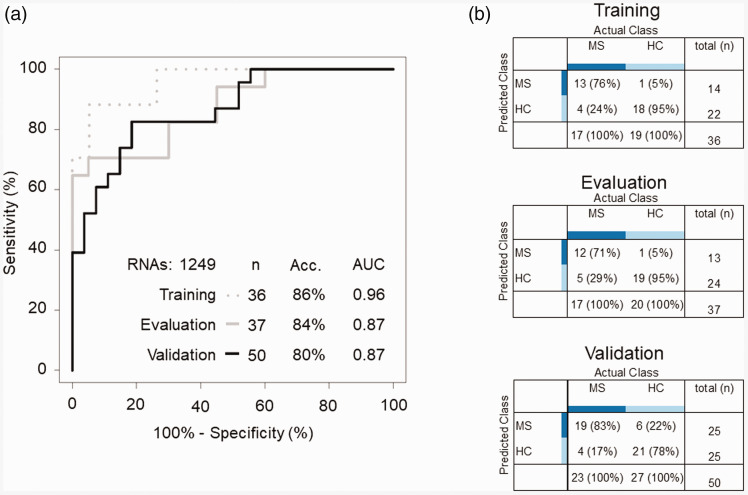
Platelet RNA profiles for MS diagnostics. (a) ROC-curve of diagnostics of healthy controls and multiple sclerosis patients. Training, evaluation and validation series are indicated separately. (b) Cross-tables of diagnostics with the optimum point from the ROC-curves. Acc = Accuracy, AUC = area under the curve.

The classification algorithm reached an accuracy of 84% (area under the curve (AUC): 0.87, 95% confidence interval (95%-CI): 0.76-0.99; Figure 2) in the evaluation series. We subsequently locked the threshold parameters of the algorithm prior to validation, employing a separate MS (n = 23) and HCs (n = 27) sample series, who were not included in algorithm development, resulting in an accuracy of 80% (AUC: 0.87 95%-CI: 0.77-0.97, sensitivity: 83%, specificity 78%; Figure 2). Post-hoc leave-one-out cross validation analysis of the training series resulted in accuracy of 86% (AUC: 0.96, 95%-CI: 0.91-1.00; Figure 2). We confirmed the sensitivity of the spliced RNA panel for the detection of MS by randomly selecting other training and evaluation series with similar sample sizes (n = 1000 iterations, median AUC: 0.89, IQR: 0.08), and confirmed the specificity of the spliced RNA panel by randomly shuffling the groups of the individual samples (n = 1000 iterations, median AUC: 0.50, IQR: 0.17).

### Discussion

We provide evidence that processes involved in MS result in alterations of platelet RNA profiles. Furthermore, we were able to demonstrate that RNA derived from circulating blood platelets may act as novel blood-based biomarker for MS. Specific splicing of platelet RNA in the presence of tumors has already been proposed in previous studies.^4,7,15^ In the case of MS, platelets appear to play an important and active role in the disease. Platelets seem to not just be involved in inflammatory and immune responses but may also contribute to the pathogenesis of MS.^10,12,16^ Here we show that blood platelets isolated from patients with MS show a distinctive RNA signature potentially of value for blood-based MS diagnostics.

We found 1249 RNA transcripts with differential levels of spliced transcripts. Compared to our previously published work regarding RNA expression levels in platelets this is a high number. Expanding the sample size will give the opportunity to set a more stringent threshold which will lower the amount of transcripts, though maintaining high accuracy. In the top-list of genes with increased expression are several proteins that have shown to play a role in activation of the immune response in MS like EPSTI1 and IFI6.^17,18^ In the top-list of genes with reduced expression RPS6KA3 is of interest, as this gene has been shown to be downregulated in blood from MS patients in remission.^19^

This study has several drawbacks. First, although we enrolled age- and gender-matched healthy controls, no individuals were included with other auto-immune or neuroinflammatory disease, potentially reducing the diagnostic accuracy. Especially Neuromyelitis Optica (NMO) would be of interest since it can mimic MS and has a different treatment approach. Second, the sample size was still small, potentially resulting in suboptimal algorithm development. Additional samples should be collected and evaluated. To reach true clinical relevance, follow-up studies should also focus on early-stage MS cases and patients presenting with a clinically isolated syndrome to assess the potential for early detection. All patients in the present study displayed a relapsing-remitting disease course. Future studies should include a broad spectrum of MS subtypes. Furthermore, additional studies are needed to gain insight into its ability to potentially predict disease progression, transition from relapsing remitting MS to secondary progressive MS, and DMT response prediction. Third, the platelet isolation method harbors a leukocyte contamination rate of 1-5 leukocytes per 1 million platelets.^7^ Though, we cannot exclude that at least a part of the profile is derived from nucleated blood cells we believe that platelet-leukocyte aggregates, which can be found in MS patients,^20^ would have an even higher mass compared to leukocytes and would therefore be eliminated during the centrifugation steps. Research focused on these aggregates could be of value.

To our knowledge, this is the first study utilizing RNA found in circulating platelets as a blood-based biomarker for distinguishing MS patients from healthy individuals. The technique’s potential for early diagnosis and treatment-response prediction, however, still need to be assessed in further studies.

## Methods

### Patients

MS patients participated in a prospective Amsterdam MS cohort study. They were included in this cohort at diagnosis and subsequently followed annually until year six and had additional follow-up at year 11. Patients have been diagnosed with MS according to the revised McDonald criteria 2017^1^ and were relapse-free and without steroid treatment for at least two months (Table 1 and Table S1). All 66 HCs were age- and gender-matched. This study was conducted in accordance with the principles of the Declaration of Helsinki. Approval of sample collection was obtained from the institutional review board and the ethics committee.

### Wet- and dry-lab procedures

Blood processing resulting in platelet RNA seq data, and subsequentially algorithm development was preformed according to previously described methods.^14^ For particle swarm-optimized (PSO) enhanced algorithm development, we applied the predefined settings; libsize correlation between −0.1 and 1.0, FDR between 0.00001 and 1.0, correlated transcripts between 0.5 and 1.0 and ranked transcripts between 200 and all detected transcripts (4,812). We selected the particle (algorithm settings) with best performance in the evaluation series following evaluation of 100 particles during 10 iterations (1000 particles in total).

## Supplemental Material

sj-xlsx-1-mso-10.1177_2055217320946784 - Supplemental material for Blood platelet RNA enables the detection of multiple sclerosis Click here for additional data file. Supplemental material, sj-xlsx-1-mso-10.1177_2055217320946784 for Blood platelet RNA enables the detection of multiple sclerosis by Nik Sol, Cyra E Leurs, Sjors GJG In ’t Veld, Eva M Strijbis, Adrienne Vancura, Markus W Schweiger, Charlotte E Teunissen, Farrah J Mateen, Bakhos A Tannous, Myron G Best, Thomas Würdinger and Joep Killestein in Multiple Sclerosis Journal—Experimental, Translational and Clinical

## Data Availability

The raw sequencing data FASTQ-files have been deposited in the NCBI GEO database.

## Code availability

The thromboSeq dry-lab pipeline is available via GitHub (https://github.com/MyronBest/thromboSeq_source_code), and is for research purposes only.
